# Editorial: Innovations and challenges in green and sustainable water purification and waste water management

**DOI:** 10.3389/fchem.2023.1235757

**Published:** 2023-06-23

**Authors:** Debasis Dhak, Agostina Chiavola, Ajay Mishra, Prasanta Dhak

**Affiliations:** ^1^ Department of Chemistry, Sidho-Kanho-Birsha University, Purulia, India; ^2^ Department of Civil, Building and Environmental Engineering, Sapienza University of Rome, Rome, Italy; ^3^ Department of Chemistry, Durban University of Technology, Durban, South Africa; ^4^ Department of Chemistry, Techno India University, Kolkata, India

**Keywords:** water purification, waste water management, challenges, green and sustainable chemical aspect, innovative techniques

Water is the essential requirement for the sustainability of living beings. The rising population and urbanization have escalated the global demand for adequate, safe, and easily available water resources. Rapid industrialization and climate change and the huge human population have led to a serious crisis of water availability and wastewater management. To overcome the crisis of sustainable water resources and wastewater management, focusing on these challenges are of the utmost importance. Currently, sustainable wastewater management is the primary focus of the Sustainable Development Goals throughout the world. Therefore, the promotion of the safe use of purified drinking water and the affordable and adequate availability of wastewater treatment techniques is the major step toward wastewater management for sustainability. The real challenge is ensuring drinking water purification by removing heavy metals, non-metals, and organic toxicants through wastewater treatment by developing green and sustainable processes and managements.

This current issue covers the use of green adsorbents, green solvents, or any green processes and technologies for the purification of drinking water and wastewater. It addresses the research and innovations, as well as the engineering and management practices, for water purification and resource restoration and recovery, thus inspiring the new generation in respect to wastewater treatment and its management through green and sustainable methods.

Liu et al. have reported the efficient removal of tetracycline from water solution by penicillin fermentation residue biochar activated by K_2_CO_3_. Microporous biochar (IKBCH) with an adequately high specific surface area was prepared by the impregnation process through HCl ageing, which has a superior adsorption effect on tetracycline. Tetracycline (TC) with a 200 mg/L, 99.91% removal rate was achieved when the microporous biochar dosage was taken at 1 g/L. The adsorption mechanisms concerned mainly the pore filling, hydrogen bonding, π-π interaction, and electrostatic adsorption. The adsorption of TC by IKBCH was very stable and would not cause secondary pollution in the environment. In addition, IKBCH had good adsorption performance on a variety of pollutants, and it was found to be a promising adsorbent.

Gradinac et al. have invented the use of titanium-made electrode in the water treatment plant of a steel plant. The electrocoagulation (EC) route was used to check the efficiency of the removal of scale ions in water using titanium-made rod electrodes. The investigation was performed on pilot electrodes. The study primarily aimed at showing the efficiency and effectiveness of the potential use of titanium-made electrodes for eliminating hardness from processed and makeup water under a closed system by utilizing a Universal Environmental Technologies system (UET). They concluded that this type of issue could be resolved with the upgradation of the UET system, maintaining the necessary equilibrium of the water, and a self-controlling system should be installed to follow the parameters, such as magnesium and calcium hardness, iron, chlorine, total hardness, corrosion, and M alkalinity and their maximum allowed limits. It is established that the efficiency and attractive water quality inside a closed system can be attained with the progression of the system with autoregulation equipment.

Biomass-based hydrogels used for the competent removal of heavy metal from water solution has been reported by Zhang et al. In this work, high-adsorption-performing hydrogel made up of porous tobacco straw combined with polyacrylic acid, STS-PAA, was prepared by pre-treated tobacco straw (TS) waste polymerized with acrylic acid/potassium acrylate by UV radiation initiation. The adsorption performance of metal ions was investigated. The effects of different temperatures, adsorption times, pH values, and initial concentrations of metal ions on the adsorption amount of heavy metal ions were investigated. The hydrogel showed a high removal rate of Pb^2+^, Hg^2+^, and Cd^2+^ in aqueous solution. The Pb^2+^ adsorption was particularly effective. At C_0_ = 4.0 mmol L^−1^, pH = 6, and the adsorption amount of Pb^2+^, Hg^2+^, and Cd^2+^ reached 1.49 mmol g^−1^, 1.02 mmol L^−1^, and 0.94 mmol g^−^, respectively, at the equilibrium condition. The kinetic data suggested that Pb^2+^, Hg^2+^, and Cd^2+^ adsorption follow the pseudo-first order model, which indicated the monolayer physical adsorption of these three heavy metal ions. A thermodynamic study established that the adsorption of Pb^2+^, Hg^2+^, and Cd^2+^ onto the STS-PAA is an endothermic (ΔH>0), entropy increase (ΔS>0), and non-spontaneous reaction.

Wang et al. have studied the effectiveness of fly ash composite filler on tailwater for the removal of nitrogen and phosphorous and its potential application on constructed wetlands (CW). This group explored 160 domestic sewage treatment facilities (DSTFs) in rural locations from two urban regions in Jiaxing, China, and found that total phosphorus (TP) and ammonia nitrogen (NH_3_-N) concentrations in rural domestic sewage (RDS) are quite high. New synthetic neutral filler (FA-SFe) was arranged from fly ash (FA) using modified acid oxidation and loaded Fe ion. When added to CW through the standard A2/O process, the highest adsorption abilities were 0.91 mg/g and 0.47 mg/g for phosphorus and nitrogen, respectively, thus improving the deficiency of TP and NH_3_-N removal. In addition, the adsorption equilibrium time of both TP and NH_3_-N by FA-SFe was low (<30 min) for the CW filler, shortening the hydraulic residence time during actual use. Therefore, FA-SFe may be used as new filler materials for CWs, minimizing nitrogen (N) and phosphorus (P) in RDS significantly.

We hope that this Research Topic has offered potential contributions, focusing light on the ways and means that will enable us to reach suitable treatment strategies and obtain safe reusable water from wastewater in a green and sustainable way.

